# Effectiveness of nutrition training of health workers toward improving caregivers’ feeding practices for children aged six months to two years: a systematic review

**DOI:** 10.1186/1475-2891-12-66

**Published:** 2013-05-20

**Authors:** Bruno F Sunguya, Krishna C Poudel, Linda B Mlunde, Prakash Shakya, David P Urassa, Masamine Jimba, Junko Yasuoka

**Affiliations:** 1 Department of Community and Global Health, Graduate School of Medicine, The University of Tokyo, 7-3-1, Hongo, Bunkyo-ku, Tokyo 113-0033, Japan; 2 Department of Public Health, School of Public Health and Health Sciences, University of Massachusetts Amherst, Arnold House, 715 North Pleasant St, Amherst, MA 01003-9304, USA; 3 School of Community and Global Health, Claremont Graduate University, 18 East Via Verde Ste. 100, Claremont, CA 91773, USA; 4 School of Public Health and Social Sciences, Muhimbili University of Health and Allied Sciences, P.O. Box 65489, Dar es Salaam, Tanzania

**Keywords:** Nutrition training, Health workers, Feeding frequency, Energy intake, Dietary diversity, Meta analysis

## Abstract

**Background:**

Nutrition training of health workers can help to reduce child undernutrition. Specifically, trained health workers might contribute to this end through frequent nutrition counseling of caregivers. This may improve child-feeding practices and thus reduce the risk of undernutrition among children of counseled caregivers. Although studies have shown varied impacts of health workers’ nutrition training on child feeding practices, no systematic review of the effectiveness of such intervention has yet been reported. Therefore, we conducted this study to examine the effectiveness of nutrition training for health workers on child feeding practices including feeding frequency, energy intake, and dietary diversity among children aged six months to two years.

**Methods:**

We searched the literature for published randomized controlled trials (RCTs) and cluster RCTs using medical databases including PubMed/MEDLINE, CINAHL, EMBASE, and ISI Web of Knowledge, and through WHO regional databases. Our intervention of interest was nutrition training of health workers. We pooled the results of the selected trials, evaluated them using the Grades of Recommendation, Assessment, Development, and Evaluation (GRADE) criteria, and calculated the overall effect size of the intervention in meta-analyses.

**Results:**

Ten RCTs and cluster RCTs out of 4757 retrieved articles were eligible for final analyses. Overall, health workers’ nutrition training improved daily energy intake of children between six months and two years of age. The pooled evidence from the three studies reporting mean energy intake per day revealed a standardized mean difference (SMD) of 0.76, 95% CI (0.63-0.88). For the two studies with median energy intake SMD was 1.06 (95% CI 0.87-1.24). Health workers’ nutrition training also improved feeding frequency among children aged six months to two years. The pooled evidence from the three studies reporting mean feeding frequency showed an SMD of 0.48 (95% CI 0.38-0.58). Regarding dietary diversity, children in intervention groups were more likely to consume more diverse diets compared to their counterparts.

**Conclusion and recommendations:**

Nutrition training for health workers can improve feeding frequency, energy intake, and dietary diversity of children aged six months to two years. Scaling up of nutrition training for health workers presents a potential entry point to improve nutrition status among children.

## Background

Undernutrition is responsible for more than one-third of child deaths globally [1], and it is more prevalent in low- and lower-middle-income countries [2]. Poverty has remained an important underlying cause of poor nutrition status among children in these regions [3,4]. It has been cited as a factor behind food insecurity [5,6], low maternal education, poor access to healthcare [7], and burden of diseases [8], each of which mediates poor child nutrition status. A disadvantaged socioeconomic position may also feed into caregivers’ poor feeding practices with regard to their children [9,10]. Poor feeding practices include low dietary diversity, feeding frequency, and energy intake [11-13].

Poor child feeding practices are caused by a myriad of factors. They are associated with cultural factors that may create local tendencies toward selection of low-quality complementary foods [14,15]; taboos [15]; and restrictive traditional beliefs [16]. Social factors including caregivers’ poor knowledge on nutrition and lack of knowledge on food diversity in their environment may correlate with poor feeding practices [17]. Such factors may result in low dietary diversity, low feeding frequency, and low food and energy intake for children.

Caregivers’ nutrition education can help to clear cultural and tradition-based misconceptions and improve their general nutrition knowledge [18]. Feeding practices can thus be improved if knowledgeable health workers treat and counsel them on proper feeding practices and monitor their progress closely [19]. Nutritionists and dieticians can, of course, perform such counseling when they are available [20-22]. However, health workers equipped with such specialized skills may not be in sufficient supply for routine care in many developing countries [23,24], leaving health workers who have only general nutrition knowledge to provide such care. Medical doctors, nurses, midwives, and midlevel providers are not always trained to perform such tasks and may not have adequate or practical knowledge to counsel and treat undernutrition [25]. Nutrition training for these cadres can help to bridge such knowledge gaps.

Available evidence supports that nutrition training of health workers can improve feeding practices and thus child undernutrition. Previous randomized controlled trials (RCTs) have found higher levels of nutrition knowledge and counseling behavior among health workers who received nutrition training [19,26,27]. Nutrition knowledge among caregivers improved in turn when they were frequently counseled by health workers who received nutrition training [19]. Nutrition counseling also improved caregivers’ knowledge in food preparation [28,29] and healthy feeding behaviors [30]. As a result, caregivers were more likely to improve their children’s feeding frequency [31], dietary diversity [32], protein, and energy intake [28,33]. Such elements of feeding practice are essential to improving children’s nutrition status [34,35].

Opportunities for routine nutrition counseling can be identified in existing health care frameworks. Globally, about 88% of pregnant mothers had at least one antenatal visit while 50% had a postnatal visit to a health care facility in 2012 [36]. These represent potential opportunities for nutrition counseling by trained health workers. Only 57% of mothers had a skilled attendant at their children’s birth [36]. However, for such mothers, there is also room for home-based nutrition counseling using community health workers or peers with relevant skills and proper nutrition training [27].

Nutrition training and counseling has also been successfully applied using specially selected and trained peer groups working hand-in-hand with trained health workers. For example, in a study conducted in Senegal, trained community health workers trained grandmothers, who in turn provided maternal and child health education on topics including child feeding to mothers and pregnant women [37]. Community and home-based interventions conducted by trained peers in collaboration with trained health workers have had success in areas with limited resources [27,38,39]. Pictures, posters, school rallies, street-side plays, and nutrition fairs have been commonly used to provide such forms of training or counseling. These illustrations can be ever more effective when tailored to the regional context [20].

Nutrition training for health workers has yielded varied impacts on child health, particularly regarding nutrition status. Previous systematic reviews have evaluated the impacts of nutrition counseling, maternal nutrition education [34], and complementary feeding on children’s nutrition status [18,40]. However, no systematic review has been conducted to evaluate the effectiveness of nutrition training for health workers on child feeding practices, an important step to improving their nutrition status. Therefore, we conducted this systematic review to examine the effect of nutrition training for health workers on feeding frequency, energy intake, and dietary diversity of children between six months and two years of age. Our PICO (Population, Intervention, Comparator, Outcome) question was as follows: “What is the effect of nutrition training for health workers to improve caregivers’ feeding practices including energy intake, feeding frequency, and dietary diversity of children under two years of age as compared to those who did not receive such training?”

## Methods

In this systematic review, we included RCTs and cluster RCTs that incorporated nutrition training for health workers as an intervention of interest. We defined nutrition training for health workers as any formal nutrition course provided to health workers in the form of in-service training, continuing professional education, short courses, or seminars, aimed, for practical or research purposes, at improving the nutrition knowledge or practices of health workers. We defined our population as health workers including doctors; nurses and nurse midwives; midlevel providers including assistant medical officers, clinical officers, assistant nurses, or assistant physicians; community health workers including village health workers; and nutritionists or dietitians working in the areas where the studies were conducted. We focused our attention on three outcome variables: feeding frequency measured in the number of times the child was fed in the previous 24 hours; energy intake in kilojoules (kJ) per day; and dietary diversity, defined as the variety of food items that was fed to a child.

We developed a review protocol based on the aforementioned guidelines that was shared among the researchers before conducting the literature search. This protocol provided guidance regarding the literature search, exclusion and inclusion criteria, methods of analysis, and grading of evidence. Based on this framework, three researchers independently conducted literature searches, selection of studies, and data extraction.

### Data sources for existing review

Three researchers independently searched the medical databases based on the review protocol. First, we searched for any existing review or submitted protocol on our topic listed in the Cochrane Library or the Cochrane Database of Systematic Reviews (CDSR). We also searched for similar review articles in the Abstracts of Reviews of Effects (DARE), National Institute for Health and Clinical Excellence (NICE), Educational Resources and Information Center (ERIC), and Campbell library of systematic reviews databases.

### Inclusion and exclusion criteria

We included RCTs and cluster RCTs that included an explanation of nutrition training provided to health workers and child feeding practices such as dietary diversity, feeding frequency, and energy intake. We also included studies that provided information and counseling on feeding practices to caregivers of children aged six months to two years.

We excluded studies that included children aged below six months and above two years. The age range of 6 months to 2 years was chosen as the focus for this study because of its importance as the transitional period from exclusive breastfeeding to complementary feeding and family foods. Studies have shown a rapid increase in rates of undernutrition during this stage [41]. Such increases in developing countries may largely be associated with inappropriate feeding practices. Also, due to wide variations in the daily required nutritional and energy intake with age, it is difficult to generalize feeding practices for children below and above two years of age. We also excluded results covering feeding practices among children on exclusive breast-feeding regimens; on special feeding interventions including parenteral feeding and tube feeding; or subject to special feeding requirements or diagnosed with chronic conditions.

### Search strategy

**Figure 1:**
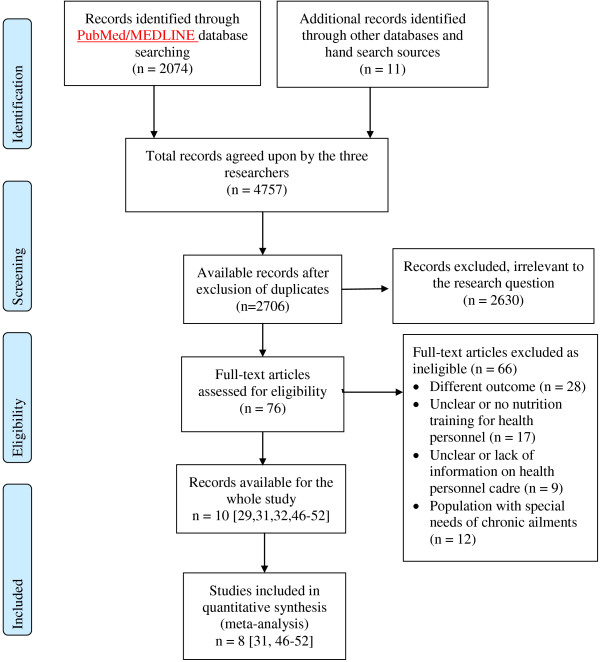
Flow diagram of information through phases of systematic review.

We conducted our literature search using medical databases including PubMed/MEDLINE, CINAHL, EMBASE, and ISI Web of Knowledge, as well as within WHO regional databases. We limited the search to a 15-year publication window (November 1st 1997 to October 30th 2012). For the PubMed databases we used the search details shown in Additional file 1. Using the Boolean combination shown in this file, we retrieved a total of 2074 articles. We used similar text words for other databases according to the database’s set-up. We also conducted hand searching using the reference lists of identified articles of interest. Figure 1 shows the flow chart of the studies thus identified and the screening process for inclusion. This figure is a modified version of the PRISMA checklist [42].

We retrieved a total of 4757 articles. Of these, 2074 articles were from PubMed and 2683 from other medical databases or retrieved through hand searches of the reference lists of initially selected articles. We compared the search results of the three independent researchers and reached agreement on the final figures. On initial screening, a total of 2051 duplicate articles were excluded because they appeared in both PubMed and other databases. Furthermore, we excluded a total of 2620 articles on initial screening which did not fit the focus of our study. We then conducted a full screening on the remaining 76 articles, out of which 66 articles were further excluded for various reasons: 17 lacked information on nutrition training of health workers, 28 had no clearly defined outcome variable matching our criteria, 9 had health personnel that did not fit our criteria of health personnel, and 12 had a population with a chronic condition or special needs (Figure 1).

### Grading of evidence and risk of bias

We evaluated the quality of the selected studies using the Grades of Recommendation, Assessment, Development, and Evaluation (GRADE) technique [43,44]. GRADE quality assessment is used to evaluate studies individually, and provides an overall assessment of the pooled evidence. The quality of evidence can be categorized as “high”, “moderate”, “low”, or “very low” based on the study design, strengths and limitations, population size, and effect size of the pooled results. Using this method, we also examined the risk of bias inherent in the individual studies included. This was also done at the outcome level.

In this study, we evaluated the quality of the evidence on energy intake and feeding frequency, arriving ultimately at a “high” quality grade (Table 1). We also conducted a risk of bias (RoB) analysis. Based on the Cochrane Handbook for Systematic Reviews of Interventions [45], five types of bias were evaluated at the study level. These were selection bias, performance bias, detection bias, attrition bias, and reporting bias. There was a serious risk of bias in two of the ten selected studies [46,47]. In Bhandari et al.’s 2001 study [46], no information on allocation concealment was available. Also, authors did not indicate whether the control group received any intervention or care, which might have introduced a performance bias. In a 2005 study by Kilaru et al. [47], meanwhile, we could not find information on allocation concealment, performance bias, or attrition bias. However, this study indicated a low risk of reporting and detection biases (Additional file 2). Despite the indicated RoB, results of these studies are not different from the rest of included studies. We therefore determined that the said risks of bias are unlikely to have markedly influenced the overall outcome of our review.

**Table 1:** Quality assessment of studies included in the systematic review

**Quality assessment**							**Summary of findings**	**GRADE quality**
No. of studies	Design	Limitations	Inconsistency	Indirectness	Imprecision	Other consideration	Effect sizes (95% CI)	[43,44]
Energy intake per day								
5	RCT and cluster RCT	No serious limitation	Three studies presented data in Mean (SD) and two in Median (IQR)	No serious indirectness	No serious imprecision	Strong association, no serious publication bias	Mean: Pooled SMD: 0.76 (0.63-0.88)	High
							Median: Pooled SMD: 1.06 (0.87-1.24)	
Feeding frequency per day								
5	RCT and Cluster RCT	No serious limitation	Three studies had Means (SD) and two had % of feeding frequency >3/day	No serious indirectness	No serious imprecision	Studies showed strong association. The pooled effective size for RR was not statistically significant.	Mean: Pooled SMD: 0.48 (0.38-0.58)	High
							Frequency: Pooled RR: 0.99 (0.87-1.13)	

### Data synthesis and meta-analysis

Outcome variables of interest in this study were energy intake, feeding frequency, and dietary diversity, all measured for the previous 24 hours. For energy intake, three studies presented the mean values and standard deviations [48-50], while two other studies presented their data in the form of median values and interquartile ranges [46,51]. Because of such diversity in the reported results, we conducted a separate meta-analysis of these studies. For consistency, we converted energy intakes in kilocalories (kcal) to kJ per day in three studies [32,50,51]. For feeding frequency, three studies presented their data as the mean feeding frequency per day [48,50,52], while two studies presented their data as percentage of participants with daily feeding frequencies greater than three [31,47]. We also conducted a separate meta-analysis due to such diversity in the reported results.

Several studies were excluded from meta-analyses due to missing or incompatible data. We did not include a study by Penny et al. [32] in meta-analyses because it lacked data on standard deviations for mean energy intake. Also, this study presented results of energy intake from animal sources only; other studies provided data on total energy intake from all complimentary foods consumed in a day. We could not conduct meta-analyses for the seven studies reporting results for the dietary diversity outcome [29,32,47-49,51,52] due to differences in the categorization used in each study and the diversity of foods available in the respective study areas.

We used the *metan* command for STATA version 12 (StataCorp, College Station, Texas, USA) to calculate the effect size in the form of standardized mean differences (SMDs) and risk ratios; to calculate their 95% CIs; to assess the statistical heterogeneity among trials; and to construct the forest plots. For the dietary diversity outcome, we compared the frequencies and percentages of foods consumed between the intervention and control group in each RCT. Though the types of foods varied across studies, they were comparable within each trial. Chi-square tests were used to compare food type consumed in intervention and control groups in the three studies.

For each study selected for analysis, we chose the comparable results reported within similar age groups for intervention and control groups to reduce the effect of heterogeneity. Some of the studies that fit our inclusion criteria also presented results of age groups beyond the set criteria. Also, some studies presented results within our age groups of interest, but followed them up for the duration of the intervention and hence presented multiple results for the same subjects. However, we selected comparable results only for children aged between 6 and 18 months. We calculated SMDs for control and intervention group within similar age groups and pooled them to calculate effect sizes for energy intake and feeding frequency. For the data presented on follow-up results, we took the results at the end of the intervention.

## Results

### Study selection

Out of 4757 identified studies, 10 studies [29,31,32,46-52] were included in the final analysis. Among these studies, 5 studies [46,48-51] had enough data for meta-analysis of energy intake and 5 studies [31,47,48,50,52] for meta-analysis of feeding frequency. None of the RCTs had suitable data for meta-analysis on dietary diversity; we therefore included 7 studies with descriptive dietary diversity data [29,32,47-49,51,52] for descriptive-only analysis of dietary diversity (Table 2).

**Table 2:** Description of studies included

**Author**	**Country**	**Design**	**Population**	**Intervention**	**Comparator**	**Results- outcome of interest**		
						**Feeding frequency**	**Energy intake**	**Dietary diversity**
Bhandari N 2001	India	RCT	Nutritionists, Caregivers and their children.	Intervention group received nutrition training from the trained nutritionists. Description of training duration for nutritionists was not provided.	Comparison group’s health workers were not trained to provide n = 106	No details	At 9 months	No details
			(Intervention-n = 104, Control 106)				• I: 978kj/day; IQR 406–1371	
							• C: 577kj/day; IQR 196–1250	
							• P < 0.05	
							At 12 months	
							• I: 1417kj/day; IQR 723–2253	
							• C: 924kj/day; IQR 474–1471	
							• P < 0.05	
Santos I 2001	Brazil	Cluster RCT	Doctors (Intervention-17, Control-16) and pairs of caregivers and their children (Intervention-218, Control-206)	Nutrition counseling component of WHO’s Integrated Management of Childhood Illness (IMCI) given for 20 hours to doctors in the intervention group. The trained doctors provided counseling to caregivers.	16 doctors did not receive nutrition counseling training. They offered general care to caregivers and their children	No details	At <18 months	Compared to Control, Intervention group had higher proportion of dietary diversity (also see Table 5)
							• Intervention: 3827.5 kJ/day; SD 1230.9	
							• Control: 3546.8 kJ/day; SD 1058.1	
							• P = 0.3	
Bhandari N 2004	India	Cluster RCT	Health and nutrition workers; pairs of caregivers and their children	Health and nutrition workers in the 4 intervention communities received nutrition training. They cared for 552 child-mother pairs.	Health and nutrition workers in 4 communities without nutrition training. They cared for 473 child-mother pairs.	At 9 months:	At 9 months:	Compared to Control, Intervention group had high proportion of dietary diversity (see Table 5)
						• I: 4.4; SD 1.5	• I: 1556 kJ/Day; SD 1109	
						• C: 3.9; SD 1.7	• C: 1025 kJ/Day; SD 866	
						At 18 months	• P < 0.01	
						• I: 5.9; SD 1.2	At 18 months	
						C: 5.4; SD 1.3	• I: 3807 kJ/Day; SD 1527	
							• C: 2577 kJ/Day; SD 1058	
Penny ME 2005	Peru	Cluster RCT	Health care workers and pairs of caregivers and their children	Health care workers in 6 health facilities received nutrition-training intervention; 187 babies were enrolled and their caregivers were counseled by these health workers.	Health care workers in 6 health facilities without the nutrition training intervention. They gave care to 190 babies enrolled in these facilities.	No details	At 9 months:	Dietary diversity at 18 months was higher in intervention group than the control group (Table 5)
							• I: 450 kcal/day	
							• C: 400 kcal/day	
							At 18 months	
							• I: 960 kcal/day	
							• C: 800 kcal/day	
							• P = 0.001	
Zaman S 2008	Pakistan	Cluster RCT	Community Health Workers and pairs of caregivers and their children	Health workers in 18 health centers received a 5 half days nutrition training using the WHO’s IMCI training module for nutrition. They recruited and gave counseling and consultation to151 child-mother pairs	Health workers in other 18 health centers without nutrition training intervention recruited and cared for 169 pairs of mothers and children	No details	• P < 0.01No details	Intervention group had a higher proportion on all the food items consumption compared to the control group (Table 5)
Shi L 2010	China	Cluster RCT	Primary healthcare providers; pairs of mothers and infants	Health care providers received nutrition training on complementary feeding, breastfeeding, and counseling skills. They counseled and provided care for 294 pairs of caregivers and their children.	Health workers from township hospitals did not receive nutrition training. Recruited and cared for 305 pairs of caregivers and their children.	At 9 months	No details	Intervention group had a higher proportion on all the food items consumed compared to the control group (Table 5)
						• I: 3.77; SD 1.62		
						• C: 2.53;		
						SD1.82		
						• P < 0.001		
						At 12 months		
						• 4.17		
						• I: 2.90; SD 1.85		
						• P < 0.001		
Vazir S 2012	India	Cluster RCT	Community health workers (Village health workers) n = 60 and 511 pairs of mothers and their children	Village health workers received supervised training on how to counsel mothers/caregivers on complementary feeding, and responsive feeding. Caregivers who received such counseling also received standard care.	Village health workers did not receive training. They provided only standard of care to caregivers and their children.	No details	At 9 months	Intervention groups (complementary and responsive feeding groups) had a higher proportion on all the food items consumption compared to the control group (Table 5)
							• I: 348 kcal/day; IQR 229,540	
							• C: 209 kcal/day; IQR 122,338	
							• P < 0.005	
							At 15 months	
							• I: 569 kcal/day; IQR 539,618	
							• C: 460; IQR 429,489	
							P < 0.005	
Roy SK 2005	Bangladesh	RCT	Nutritionists, medical officer, and health assistants	Two-week nutrition training was conducted for health workers. The training included nutrition education, counseling, and anthropometry. Trained health workers provided counseling to mothers of moderately malnourished children on complementary feeding.	Mothers of a control group received normal care from health workers who received no nutrition training	Feeding frequency >3 times/day	No details	No details
						At 3 months		
						I: 98%, C: 54%		
						At 6 months		
						I: 97%, C: 58%		
Pachon H 2002	Vietnam	Cluster RCT	Community health workers and 240 pairs of caregivers and their children	Training implementers (health workers) who are also health volunteers received nutrition training to implement intensive nutrition rehabilitation sessions for ten months. Counseling for caregivers was done twice a week for nine months.	Health workers were not trained to implement intensive nutrition rehabilitation sessions.	At 2–6 months	At 2–6 months	No details
						• I: 4.6; SD 1.3	• I: 662.7 kcal/day; SD 301.0	
						• C: 4.2; SD 1.1	• C: 597.4 kcal/day; SD 275.7	
						• P < 0.01	• P < 0.1	
						At 12 months	At 12 months	
						• I: 4.9; SD 1.5	• I: 826.9 kcal/day SD 324.4	
						• C: 4.4; SD 1.5	• C: 718.4 kcal/day SD 330.0)	
						P < 0.01	• P < 0.01	
Kilaru A 2005	India	Cluster RCT	Auxiliary nurse midwives, community health workers	Auxiliary nurse midwives, community health workers received nutrition training from MCH consultant (pediatrician and nutritionists). They provided counseling to 173 caregivers and their children	Normal standard of care provided by auxiliary nurse midwives who did not receive any special nutrition training	At 7-11 months Feeding frequency >4 times/day	No details	At 11 months
						• I: 78%		Feeding at least 5 types/day
						• C: 51%		• I: 42%
						• P < 0.001		• C: 19%
								P = 0.01

Footnotes.

*I* - Intervention group, *C* - Control group, *P* - P value, *SD* - Standard deviation, *IQR* - Inter quartile range, *CI* - Confidence Interval.

### Energy intake per day for children aged six months to two years

The pooled evidence from the three trials reporting on mean energy intake (Table 3) showed that, nutrition training of health workers improved daily energy intake of children aged six months to two years. The pooled SMD between the intervention and control groups was 0.76 with a 95%CI of 0.63 to 0.88 in a random model. The test for overall effect gave z = 12.17, P < 0.001. For the two studies with median energy intake presented with interquartile ranges (Table 3), the pooled SMD was 1.06, with a 95% CI of 0.87 to 1.24. The test for overall effect gave z = 11.22, P < 0.001.

**Table 3:** Effectiveness of the intervention on the energy intake per day mean energy intake (kJ/day)

**Study, year**	**SMD**	**95% CI**	**% Weight**	**Forest plot**
Bhandari N, 2004	0.93	0.78-1.07	72.07	
Santos I, 2001	0.24	−0.24-0.73	6.32	
Pachon H, 2002	0.33	0.07-0.59	21.61	
I-V Pooled SMD	0.76	0.63-0.88		
Heterogeneity chi-square = 19.91 (d.f. = 2) p < 0.001				
Test of SMD = 0; z = 12.17 p < 0.001				
**Median energy intake (kJ/day)**				
Bhandari N, 2001	0.38	0.10-0.67	42.45	
Vazir S, 2012	1.55	1.31-1.80	57.55	
I-V pooled SMD	1.06	0.87-1.24		
Heterogeneity chi-squared = 37.63 (d.f. = 1) p <0.001				
Test of SMD = 0: z = 11.22 p < 0.001				

### Feeding frequency for children aged six months to two years

The pooled evidence from the three trials reporting on mean feeding frequency (Table 4) showed that nutrition training of health workers improved feeding frequency of children under two years of age. The SMD between the intervention and control groups was 0.48, with a 95% CI of 0.38 to 0.58 in a random model. The test for overall effect gave z = 9.17, P < 0.001. For the two studies with feeding frequency as a percentage (Table 4) showed pooled risk ratio (RR) of 0.99, 95% CI (0.87-1.13). The test for overall effect, z = 0.09, P = 0.926.

**Table 4:** Effectiveness of intervention on the feeding frequency per day mean feeding frequency per day

**Study, year**	**SMD**	**95% CI**	**% Weight**	**Forest plot**
Bhandari N, 2004	0.40	0.26-0.54	55.94	
Shi L, 2009	0.72	0.53-0.91	28.82	
Pachon H, 2002	0.33	0.07-0.60	15.24	
I-V Pooled SMD	0.48	0.38-0.58		
Heterogeneity chi-squared = 8.42 (d.f. = 2) p = 0.015				
Test of SMD = 0 : z = 9.17 p <0.001				
**Feeding frequency >3 times per day**				
Study, year	RR	95% CI	% Weight	
Roy SK, 2005	0.80	0.66-0.98	52.01	
Kilaru A, 2005	1.20	1.01-1.43	47.99	
M-H pooled RR	0.99	0.87-1.13		
Heterogeneity chi-squared = 8.93 (d.f. = 1), p = 0.003				
Test of RR = 1: z = 0.09, p = 0.926				

### Dietary diversity for children aged six months to two years

In the RCT conducted in Brazil [49], health workers who received nutrition training provided counseling to caregivers. Children whose caregivers were counseled by trained health workers had a higher dietary diversity compared to their counterparts (P < 0.001). Similar interventions were conducted in India [47,48,51]. In these trials, too, children in intervention groups had significantly higher dietary diversities compared to their counterparts in control groups. Other RCTs conducted in Pakistan [29], Peru [32], and China [52] showed similar results: high percentages of children in intervention group consumed each type of food items compared to their counterparts in the control group (Table 5).

**Table 5:** Effectiveness of the intervention on the dietary diversity of children under two years of age

**Author, Year**	**Outcome**	**Intervention (%)**	**Control (%)**	**P-value**
1. Santos I, 2001	Dietary diversity at 18 months	N = 206	N = 216	P-value
	Egg yolk	19.20	8.20	P < 0.01
	Shredded chicken and beef	15.50	6.30	P < 0.01
	Chicken liver	20.50	6.80	P < 0.001
	Oil, margarine or butter	16.90	0.50	P < 0.001
2. Bhandari N, 2004	Dietary diversity at 18 months	N = 435	N = 394	
	Cereal legume gruel or mix	49.6	31.7	P < 0.001
	Milk cereal gruels or mix	133.3	14.9	P < 0.001
	Undiluted milk	60.5	12.9	P < 0.001
	Added oil/butter	24.1	5.8	P < 0.001
	Snacks	58.2	54.1	
	Commercially available bread	23.0	10.7	P < 0.001
	Home-made bread	82.1	86.3	
	Rice	8.3	7.6	
	Potatoes	29.0	22.1	P < 0.001
	Legumes	29.7	23.9	P < 0.01
	Milk	98.6	95.9	
	Vegetables	26.0	24.1	
	Fruits	144.8	40.4	
3. Penny ME, 2005	Dietary diversity at18 months	N = 171	N = 167	
	Egg, chicken liver or fish	64.0	57.0	
4. Zaman S, 2008	Dietary diversity at 18 months	N = 126	N = 131	
	Eggs	47.6	26.7	
	Chicken/beef/mutton	60.3	39.7	
	Liver	30.9	19.9	
	Added ghee/butter/oil	53.9	38.2	
	Thick kitchuri	65.9	44.3	
5. Shi L, 2010	Dietary diversity at 12 months	N = 256	N = 234	
	Bread, rice, noodles	100	98.3	
	Roots or tubers	90.9	73.8	
	Yellow/orange foods	97.2	76.7	
	Green leafy vegetables	97.6	87.9	
	Beans/peas/lentils	92.1	67.2	
	Fruits	99.6	96.6	
	Eggs	98.8	92.2	
	Meat or organ meats	96.9	58.2	
	Cooking oils/fats	96.5	79.7	
6. Kilaru G, 2005	Dietary diversity at 11 months	N = 173	N = 69	
	At least 5 different groups	42.0	19.0	P = 0.01
7. Vazir S, 2012	Dietary diversity at 15 months	N = 170	N = 168	
	Rice	99.5	94.9	
	Goat/chicken liver	38.0	13.1	
	Goat meat	43.5	33.0	
	Poultry	37.5	18.9	
	Banana	79.3	61.9	
	Buffalo milk	81.5	72.7	
	Egg	73.9	54.0	
	Spinach	42.4	29.5	
	Pulses	89.7	71.6	
	Added fat	42.4	29.5	

## Discussion

This is the first systematic review to evaluate the effectiveness of nutrition training of health workers on child feeding practices. Previous reviews showed the effectiveness of maternal nutrition education and complimentary feeding interventions to improve child feeding practices [18] and nutrition status [34,40]. Our study helps to show a possible pathway to improve child nutrition status by starting with health worker training. We found that training of health workers can help to improve feeding practices of children between six months and two years of age. The children whose caregivers were counseled by the trained health workers had a higher mean feeding frequency, energy intake, and dietary diversity compared to their counterparts.

Strong evidence thus suggests that nutrition training of health workers improves energy intake, feeding frequency, and dietary diversity of children between six months and two years of age. Such a significant outcome may be conceived of through the following pathway: First, nutrition training can increase or refresh health workers’ nutrition and food sciences-related knowledge. Indeed, two RCTs conducted in Brazil [19] and India [46] found that nutrition training of health workers improved their knowledge in nutrition. Nutrition training can be used to update health workers’ nutrition knowledge and to alert them to new findings pertinent to their environments [16,28,29,53]. This will enable them to address determinants of undernutrition specific to their areas, and to improve their communication, counseling, and undernutrition management skills [19,29,32,48,49]. Updated management skills including tailored counseling may also be important for the effective transfer of knowledge to the end users – in this case, the caregivers.

Second, nutrition knowledge transfer by skilled and trained health workers may be achieved when they counsel caregivers who visit health facilities [20]. Similarly, trained health workers may also access caregivers through outreach and home visits even in rural areas, and may achieve a similar outcome through such routes [47,54]. Previous RCTs showed improved nutrition knowledge and knowledge retention among caregivers counseled by health workers who received nutrition training [16,29,31,32,49,54].

Third, the counseled caregivers can serve as agents of change. Caregivers endowed with updated nutrition knowledge through frequent counseling can improve their own child feeding behaviors [16,20,28,29,31,32,47-49,52],[55]. Such behaviors may include food preparation hygiene, feeding frequency, proper mixing of quality foods, increased energy intake, and dietary diversity. Thus, children’s growth can improve and their risk of undernutrition can be minimized [34]. Secondarily, other determinants of undernutrition such as food-borne infections can be reduced [32,56,57] and food preparation hygiene improved [52].

Nutrition counseling from trained health workers has been proven effective even in areas of limited food availability [31,34]. In such circumstances, caregivers were able to choose the right mix of foods under availability constraints. For example, in the RCT conducted in Bangladesh, about a third of families were poor and lived in food-insecure households. Despite such hardship, nutrition knowledge gained from trained health workers motivated and changed their feeding behavior. Thus, they could provide the required balance of foods to their children [31].

The findings of this review should be interpreted in light of several limitations. First, the selected studies came from different regions and there is a risk of regional variations. Such regional variations can cause differences in characteristics of participants as shown in Table 2. Also, the selected studies were conducted in the context of different health systems. In this case, the nutrition training was conducted to the health workers of different carders. For example, in Bangladeshi and India studies, training was conducted among nutritionists and other health carders including medical officers. In other settings, training was conducted among health carders available in such settings, including doctors, primary health care providers, auxiliary nurses, midwives, health assistants, and community health workers as shown in Table 2. To minimize this limitation, we selected RCTs and cluster RCTs as these studies can minimize the effect that could have been caused by differences in intervention and control groups. Meta-analysis pools the SMDs of each study into a single effective size. This can help to reduce any discrepancies arising from variations across studies.

Second, we could not conduct a meta-analysis for the dietary diversity outcome. This was due to the differences in types of foods reported in the trials included in this study. Such differences were also due to regional variations in the typical diet. Also, in all the selected studies, dietary diversity was not a primary outcome. Lack of a standard method for data collection on dietary diversity might also be a reason for such differences. To minimize the effect of variations in food type, regional, and methodological aspects, we compared the results of diets consumed within the trials. All the seven trials showed better dietary diversity for the intervention compared to the control groups. Therefore, despite the regional and methodological differences in reporting dietary diversity, all studies showed the effectiveness of the intervention on dietary diversity among children under two years of age.

Third, our results showed a significant heterogeneity among the selected studies. This might manifest in differences in training duration and qualifications of health workers, in targeted age groups, in follow-up procedures, and in regional context. We could not retrieve the training duration for all of the selected trials. However, some of the selected trials used a standardized Integrated Management of Childhood Illness (IMCI) training manual developed by WHO, while others used results of formative research conducted prior to the trial. Moreover, results from all the selected studies were consistent. Although these studies were conducted in different regional contexts, they all showed a significant improvement in feeding practices when health workers received nutrition training.

Fourth, due to time limitations, we did not register a study protocol prior to the review process. To minimize such limitations, we developed the in-house review protocol based on the pre-set guidelines before starting the evidence search. The protocol was shared among the research team and the three independent researchers who conducted the evidence search. We evaluated each step of data collection as a team to verify the scrupulous use of the protocol. To this end, we were satisfied that the original protocol was adhered to.

Fifth, our results may also not be generalizable beyond the low- and middle-income countries where the selected studies were conducted. However, based on the global nutrition situation, these are the areas with the highest burden of child undernutrition. These results may thus be especially useful to scale up the nutrition training of health workers toward improving the current child undernutrition situation.

Despite its limitations, our study also has notable strengths. This is the first systematic review to examine the effectiveness of nutrition training of health workers on child feeding practices. Second, we used the GRADE method to critically assess the quality and strength of the evidence presented. Overall, the evidence of intervention effectiveness on feeding frequency and energy intake was of high quality. Thus, the results of this systematic review may help to design policies to improve feeding practices of children through training of available health workforce cadres.

In conclusion, nutrition training for health workers can improve feeding practices for children under two years of age. Such practices include feeding frequency, energy intake, and dietary diversity. Training materials should be prepared based on the local context and should include information on how to identify foods that are available, affordable and acceptable, which is particularly important in areas of limited food availability. Moreover, trained health workers offer the prospect of an accessible and reliable information resource for local families. In this way, nutrition training for health workers can serve as an important entry point for a sustainable strategy toward improving the nutrition status of young children.

## Competing interest

The authors declare that they have no competing interest.

## Authors’ contributions

BFS conceived the research questions, designed the study, participated in the literature review and analyses, and prepared the first draft. KCP refined the research question, participated in analysis, and refined the first draft. LBM contributed to the study design, participated in the literature review and analyses, and helped to prepare the first draft. PS participated in the literature review and analysis. DPU revised the protocol and the drafted manuscript. MJ reviewed the study protocol, the manuscript, and approved the submission. JY participated in the research design, analyses, and preparation of the first draft and revisions. All the authors read and approved the final version of the manuscript for submission.

## Supplementary Material

Additional file 1 Search strategy: PubMed. Click here for file

Additional file 2 Risk of bias assessment. Click here for file
