# Supplementary material for: Effectiveness of nutrition training of health workers toward improving caregivers’ feeding practices for children aged six months to two years: a systematic review
Source: Nutr J. 2013 May 20;12:66. doi: 10.1186/1475-2891-12-66 (PMC3668136; doi:10.1186/1475-2891-12-66)
Supplement: Additional file 1 — Search strategy: PubMed. [file 1475-2891-12-66-S1.doc]

**Additional file 1**

**Search strategy: PubMed**

**Search terms used:**

**Population**: Caregivers [MeSH Terms] OR caregivers [All Fields] OR parents [MeSH Terms] OR parents [All Fields]

**OR**

Health personnel [MeSH Terms] OR (health [All Fields] AND personnel [All Fields]) OR health personnel [All Fields]

**Intervention:** nutritional status [MeSH Terms] OR (nutritional [All Fields] AND status [All Fields]) OR nutritional status [All Fields] OR nutrition [All Fields] OR nutritional sciences [MeSH Terms] OR (nutritional [All Fields] AND sciences [All Fields]) OR nutritional sciences [All Fields]

**AND**

Education [Subheading] OR education [All Fields] OR training [All Fields] OR education [MeSH Terms] OR training [All Fields] OR (inservice training [MeSH Terms] OR inservice [All Fields] AND training [All Fields] OR inservice training [All Fields]

**Outcome:** Nutritional sciences [MeSH Terms] OR (nutritional [All Fields] AND sciences [All Fields]) OR nutritional sciences [All Fields] OR (nutrition [All Fields] AND sciences [All Fields]) OR nutrition sciences [All Fields]

**OR**

Feeding behavior [All Fields] OR feeding behavior [MeSH Terms] OR (feeding [All Fields] AND behavior [All Fields]) OR feeding behavior [All Fields]

**OR**

Diet [MeSH Terms] OR diet [All Fields]) OR diet [MeSH Terms] OR diet [All Fields] OR dietary [All Fields] OR (dietary [All Fields] AND diversity [All Fields]) OR (feeding [All Fields] AND (epidemiology [Subheading] OR epidemiology [All Fields]))

**OR**

Feeding [All Fields] AND (frequency [All Fields] OR epidemiology [MeSH Terms] OR frequency [All Fields])

**AND**

Dates of publication between 1997/11/01 to 2012/10/30

The combination was made as follows:

Population terms **AND** Intervention terms **AND** Outcome terms **AND** dates of publication

**The resulted Boolean combination for PubMed was therefore:**

((((("caregivers"[MeSH Terms] OR "caregivers"[All Fields] OR "caregiver"[All Fields]) OR ("parents"[MeSH Terms] OR "parents"[All Fields])) OR ("health personnel"[MeSH Terms] OR ("health"[All Fields] AND "personnel"[All Fields]) OR "health personnel"[All Fields])) AND ((((("nutritional status"[MeSH Terms] OR ("nutritional"[All Fields] AND "status"[All Fields]) OR "nutritional status"[All Fields] OR "nutrition"[All Fields] OR "nutritional sciences"[MeSH Terms] OR ("nutritional"[All Fields] AND "sciences"[All Fields]) OR "nutritional sciences"[All Fields]) AND ("education"[Subheading] OR "education"[All Fields] OR "training"[All Fields] OR "education"[MeSH Terms] OR "training"[All Fields])) OR ("inservice training"[MeSH Terms] OR ("inservice"[All Fields] AND "training"[All Fields]) OR "inservice training"[All Fields])) OR (("nutritional status"[MeSH Terms] OR ("nutritional"[All Fields] AND "status"[All Fields]) OR "nutritional status"[All Fields] OR "nutrition"[All Fields] OR "nutritional sciences"[MeSH Terms] OR ("nutritional"[All Fields] AND "sciences"[All Fields]) OR "nutritional sciences"[All Fields]) AND ("inservice training"[MeSH Terms] OR ("inservice"[All Fields] AND "training"[All Fields]) OR "inservice training"[All Fields]))) OR (("nutritional status"[MeSH Terms] OR ("nutritional"[All Fields] AND "status"[All Fields]) OR "nutritional status"[All Fields] OR "nutrition"[All Fields] OR "nutritional sciences"[MeSH Terms] OR ("nutritional"[All Fields] AND "sciences"[All Fields]) OR "nutritional sciences"[All Fields]) AND ("education"[Subheading] OR "education"[All Fields] OR "educational status"[MeSH Terms] OR ("educational"[All Fields] AND "status"[All Fields]) OR "educational status"[All Fields] OR "education"[All Fields] OR "education"[MeSH Terms])))) AND (((((("nutritional sciences"[MeSH Terms] OR ("nutritional"[All Fields] AND "sciences"[All Fields]) OR "nutritional sciences"[All Fields] OR ("nutrition"[All Fields] AND "sciences"[All Fields]) OR "nutrition sciences"[All Fields]) OR ("feeding behaviour"[All Fields] OR "feeding behavior"[MeSH Terms] OR ("feeding"[All Fields] AND "behavior"[All Fields]) OR "feeding behavior"[All Fields])) OR ("diet"[MeSH Terms] OR "diet"[All Fields])) OR ("diet"[MeSH Terms] OR "diet"[All Fields] OR "dietary"[All Fields])) OR (("diet"[MeSH Terms] OR "diet"[All Fields] OR "dietary"[All Fields]) AND "diversity"[All Fields])) OR (feeding[All Fields] AND ("epidemiology"[Subheading] OR "epidemiology"[All Fields] OR "frequency"[All Fields] OR "epidemiology"[MeSH Terms] OR "frequency"[All Fields])))) AND ("1997/11/01"[PDAT]: "2012/10/30"[PDAT]).
