# Supplementary material for: Effectiveness of nutrition training of health workers toward improving caregivers’ feeding practices for children aged six months to two years: a systematic review
Source: Nutr J. 2013 May 20;12:66. doi: 10.1186/1475-2891-12-66 (PMC3668136; doi:10.1186/1475-2891-12-66)
Supplement: Additional file 2 — Risk of bias assessment. [file 1475-2891-12-66-S2.doc]

Additional File 2

Risk of bias assessment

| Bhandari N 2001 | Santos I 2001 | Bhandari N 2004 | Penny ME 2005 | Zaman S 2008 | Shi L 2010 | Roy SK 2005 | Vazir S 2010 | Pachon H 2002 | Kilaru A 2005 |  |
| --- | --- | --- | --- | --- | --- | --- | --- | --- | --- | --- |
| B | A | A | A | A | A | A | A | A | C | Selection bias: Allocation concealment |
| C | A | A | B | A | A | A | A | A | B | Performance bias: Blinding of participants and personnel |
| A | A | A | A | A | A | A | A | A | A | Detection bias: Blinding of outcome assessment |
| A | A | A | A | A | A | B | A | A | B | Attrition bias: Incomplete outcome data |
| A | A | A | A | A | A | A | A | A | A | Reporting bias: Selective reporting |

A: Low risk of bias B: Unclear risk of bias C: High risk of bias
